# Development of coronary heart disease and ischemic stroke in relation to fasting and 2-hour plasma glucose levels in the normal range

**DOI:** 10.1186/1475-2840-11-76

**Published:** 2012-06-25

**Authors:** Feng Ning, Lei Zhang, Jacqueline M Dekker, Altan Onat, Coen DA Stehouwer, John S Yudkin, Tiina Laatikainen, Jaakko Tuomilehto, Kalevi Pyörälä, Qing Qiao

**Affiliations:** 1 Department of Public Health, Hjelt Institute, University of Helsinki, Helsinki, Finland; 2 Department of Chronic Disease Prevention, National Institute for Health and Welfare, Helsinki, Finland; 3 Qingdao Endocrine & Diabetes Hospital, Qingdao, China; 4 Weifang Medical University, Weifang, China; 5 Department of Epidemiology and Biostatistics EMGO, Institute for Health and Care Research VU University Medical Center, Amsterdam, the Netherlands; 6 Turkish Society of Cardiology, Istanbul, Turkey; 7 Department of Cardiology, Cerrahpaşa Medical Faculty, Istanbul University, Istanbul, Turkey; 8 Department of Internal Medicine and Cardiovascular Research Institute Maastricht, Maastricht University Medical Centre, Maastricht, the Netherlands; 9 University College London, WC1E 6BT, London, UK; 10 South Ostrobothnia Central Hospital, Seinäjoki, Finland; 11 Red RECAVA Grupo RD06/0014/0015, Hospital Universitario La Paz, Madrid, Spain; 12 Institute of Clinical Medicine, Faculty of Health Sciences, University of Eastern Finland, Kuopio, Finland

**Keywords:** Normoglycemia, Coronary heart disease, Ischemic stroke, Incidence

## Abstract

**Background:**

Individuals who had normoglycemia but whose 2-hour plasma glucose (2hPG) concentrations did not return to the fasting plasma glucose (FPG) levels during an oral glucose tolerance test (OGTT) have been shown to have increased cardiovascular mortality. This is further investigated regarding to the first events of coronary heart disease (CHD) and ischemic stroke (IS).

**Method:**

Data from 9 Finnish and Swedish cohorts comprising 3743 men and 3916 women aged 25 to 90 years who had FPG < 6.1 mmol/l and 2hPG < 7.8 mmol/l and free of CVD at enrolment were analyzed. Hazard ratios (HRs) for first CHD and IS events were estimated for the individuals with 2hPG > FPG (Group II) compared with those having 2hPG ≤ FPG (Group I).

**Results:**

A total of 466 (115) CHD and 235 (106) IS events occurred in men (women) during a median follow-up of 16.4 years. Individuals in Group II were older and had greater body mass index, blood pressure, 2hPG and fasting insulin than those in Group I in both sexes. Multivariate adjusted HRs (95% confidence intervals) for incidence of CHD, IS, and composite CVD events (CHD + IS) in men were 1.13 (0.93-1.37), 1.40 (1.06-1.85) and 1.20 (1.01-1.42) in the Group II as compared with those in the Group I. The corresponding HRs in women were 1.33 (0.83-2.13), 0.94 (0.59-1.51) and 1.11 (0.79-1.54), respectively.

**Conclusion:**

Within normoglycemic range individuals whose 2hPG did not return to their FPG levels during an OGTT had increased risk of CHD and IS.

## Background

The role of hyperglycemia on the mortality from coronary heart disease (CHD) [1,2], stroke [3,4] and other cardiovascular diseases (CVDs) [5-7] has been well investigated. Two-hour plasma glucose (2hPG) is a better predictor than fasting plasma glucose (FPG) for incidence of CHD [8,9] and ischemic stroke (IS) [10] among individuals with hyperglycemia, but little is known about their impact within normoglycemic range. Previous studies have shown that insulin resistance and declined beta cell function are already presented in subjects with elevated normal FPG and/or 2hPG [11-13]. The upper normal level of the FPG [14,15] or 2hPG [16] has been found to increase the risk of type 2 diabetes. Our previous finding based on the DECODE (Diabetes Epidemiology: Collaborative analysis Of Diagnostic criteria in Europe) study demonstrated that individuals with normoglycemia, whose 2hPG did not return to the FPG levels during a standard 75 g 2-h oral glucose tolerance test (OGTT) had a higher risk for mortality from CVD and all-cause than individuals whose 2hPG returned to their FPG levels or below them [17]. However, the early DECODE study could not show a causal relationship because individuals with CVD at baseline could not be identified and excluded. The elevated 2hPG could be a consequence of existing CVD rather than a cause. To further clarify the causal relationship between elevated normal 2hPG and the development of the CVD events, the current study is carried out based on the Finnish and the Swedish DECODE cohorts who have complete information on the occurrence of both fatal and non-fatal events.

### Participants and methods

Data from 9 Finnish and Swedish cohorts comprising 3743 men and 3916 women aged 25 to 90 years who participated in the DECODE study were collaboratively analyzed. The maximum duration of follow-up ranged from 12.4 to 36.8 years among different cohorts with a median follow-up of 16.4 years. The study populations and methods used to recruit participants in the DECODE study had been described previously [18-21]. Briefly, the database was collected from researchers in Europe who had performed epidemiological surveys for diabetes using a standard 2-h OGTT. Individuals’ data from participating study cohorts were sent to the Department of Chronic Disease Prevention of the National Institute for Health and Welfare in Helsinki, Finland for data analyses. In the current study, only the cohorts with the prospective data on fatal and non-fatal events with all required covariates of body mass index (BMI), blood pressure, total serum cholesterol and smoking status were included. Each study had been approved by the local ethics committees, and the ethics committee of the National Institute for Health and Welfare approved the collaborative data analysis.

BMI was calculated as weight in kilograms divided by height in meters squared (kg/m^2^). An individual with a prior history of hypertension or having a systolic blood pressure (SBP) ≥ 140 mmHg and/or a diastolic blood pressure (DBP) ≥ 90 mmHg and/or receiving antihypertensive treatment was defined as having hypertension [22]. To reduce the bias derived from differences in methodology between studies, cohorts-specific Z-score (Z = [X-μ]/σ) was calculated for fasting insulin concentrations. Insulin resistance was estimated by the homeostasis model assessment according to formula of HOMA-IR = (fasting insulin* FPG)/22.5 [23]. The smoking status was classified as current smoker, ex-smoker or non-smoker.

Individuals with a prior history of diabetes or newly diagnosed diabetes (FPG ≥ 7.0 mmol/l and/or 2hPG ≥ 11.1 mmol/l), or pre-diabetes (FPG 6.1-6.9 mmol/l and/or 2hPG 7.8-11.0 mmol/l) at baseline were excluded from the current data analysis. The data analysis was restricted to the normoglycemic individuals defined by a FPG < 6.1 mmol/l and a 2hPG < 7.8 mmol/l according to the World Health Organization and International Diabetes Federation 2006 criteria [24]. The normoglycemic individuals were further classified into Group I if the 2hPG after a 75 g oral glucose load was equal to or less than the FPG (2hPG ≤ FPG), or group II if the 2hPG was greater than the FPG (2hPG > FPG).

### Definition of CHD and IS events

First-ever CHD and stroke events occurred before the 31 December 2008 were ascertained through computerized record linkage of the unique national identification numbers of the survey participants to the national Causes of Death Registry and the national Hospital Discharge Registry in both Finnish [25,26] and Swedish [27] studies. Individuals with a history of CHD, myocardial infarction, or stroke diagnosed before the baseline examination were excluded from the current data analysis. Incident CHD events were defined as fatal CHD or non-fatal myocardial infarction; IS events were defined as fatal or non-fatal IS. The International Classification of Diseases (ICD), 9th Revision (10th Revision), were used for the classification of either fatal or non-fatal events: ICD codes 410 to 414 (I20 to I25) for fatal CHD; 410 to 411 (I21 to I22, I24) for non-fatal acute myocardial infarction; 433, 434, 436 (I63 to I64) for fatal and non-fatal IS. A composite CVD event consists of first events of both CHD and IS.

### Statistical analysis

A general linear model was used to estimate the mean of a continuous variable adjusting for age and cohort. The Chi-square test was performed to assess the difference in proportions between groups. Interaction between sex and glucose groups was examined. The Schoenfeld-test was employed to assess the proportional hazards assumption, and the results showed the proportionality assumption was not violated. Cox proportional hazards analysis was employed to estimate hazard ratios (HRs) for CHD, IS incidence and composite CVD events in the Group II as compared with the Group I. In the multivariate Cox model, we adjusted for age, cohort, BMI, FPG, total serum cholesterol, smoking status and hypertension status. The difference between 2hPG and FPG as a continuous variable (2hPG-FPG) was also fitted in a separate multivariate model adjusting for the baseline FPG to examine whether the relationship was linear. In addition, to check whether the “return of the 2hPG to the FPG level” was determined by the FPG levels, the comparison of Group II versus Group I was further assessed by dividing the FPG into two strata of FPG ≤ 5.6 mmol/l and FPG 5.6-6.1 mmol/l. The statistical analyses were performed using SPSS version 18.0 software (SPSS Inc. Chicago, USA) and STATA version 11.0 software (Stata Corp., TX, USA). A p-value less than 0.05 (two sided tests) was considered statistically significant.

## Results

Baseline characteristics of the participants are presented in Table 1. During a median follow-up of 16.4 years, a total of 466 (115) CHD, 235 (106) IS and 638 (213) composite CVD events in men (women) were recorded. The incidence of IS, composite CVD events were higher in the Group II than in the Group I in men (p < 0.05), but not in women (Table 2). Both incidences of CHD (7.2 per 1000 person-years versus 1.8 per 1000 person-years) and IS (3.7 per 1000 person-years versus 1.6 per 1000 person-years) were higher in men than in women (p < 0.05 for all comparisons). In addition, mean values of various CVD risk factors such as age, BMI, 2hPG, blood pressure, fasting insulin and HOMA-IR were significantly greater in individuals in the Group II than in those in the Group I in both sexes (p < 0.05 for all comparisons), except for BMI and systolic blood pressure in women.

**Table 1:** Baseline characteristics of study cohorts and number of first events of CHD and IS

**Countries and studies**	**No. (men/women)**	**Age years Mean (range)**	FPG^*^ (mmol/l)	2hPG^*^ (mmol/l)	**No. of events (men/women)**		**Maximum (Median) follow-up years**
					**CHD**	**IS**	
Finland							
East–West Study	125/-	76 (70–90)	5.2 (0.04)	5.4 (0.10)	27/-	24/-	16.8 (10.4)
FINRISK 1987	859/1006	53 (44–64)	5.1 (0.01)	5.5 (0.03)	142/59	57/46	21.0 (20.8)
FINRISK 1992	496/747	53 (44–64)	5.2 (0.01)	5.5 (0.03)	32/18	8/11	16.0 (15.9)
Helsinki Policemen Study	578/-	45 (31–69)	5.7 (0.02)	5.3 (0.05)	141/-	77/-	36.8 (34.5)
Oulu Study	93/172	55 (55–55)	5.4 (0.03)	6.1 (0.07)	4/4	5/5	17.3 (16.8)
Vantaa Study	120/168	65 (64–66)	5.2 (0.03)	6.2 (0.07)	16/15	14/9	17.9 (17.3)
Sweden							
Malmö Prevention Project	-/821	54 (48–57)	5.5 (0.02)	6.7 (0.04)	-/2	-/16	20.0 (14.6)
MONICA	923/1002	45 (25–74)	5.1 (0.01)	5.3 (0.03)	50/17	12/19	20.6 (16.4)
ULSAM	549/-	71 (70–73)	5.1 (0.02)	5.5 (0.06)	54/-	38/-	12.4 (10.2)
Total	3743/3916	53 (25–90)	5.2 (0.01)	5.6 (0.01)	466/115	235/106	36.8 (16.4)

^*^Age-adjusted means (standard error). FPG, fasting plasma glucose; 2hPG, two-hour plasma glucose; CHD, coronary heart disease; IS, ischemic stroke; MONICA, Monitoring of trends and determinants in cardiovascular disease; ULSAM, the Uppsala Longitudinal Study of Adult Men study.

**Table 2:** Characteristics of participants and incidence of CHD and IS according to FPG and 2hPG categories

	**Men**		**Women**	
	**Group I**	**Group II**	**Group I**	**Group II**
	**2hPG ≤ FPG**	**2hPG > FPG**	**2hPG ≤ FPG**	**2hPG > FPG**
no. (%)	1818 (48.6)	1925 (51.4)	1110 (28.3)	2806 (71.7)
Age (years)	50 (0.28) ^*^	57 (0.27)	49 (0.25) ^*^	53 (0.16)
Body mass index (kg/m^2^)	25.9 (0.08) ^*^	26.3 (0.07)	25.6 (0.13)	25.9 (0.08)
FPG (mmol/l)	5.33 (0.01) ^*^	5.18 (0.01)	5.23 (0.01) ^*^	5.18 (0.01)
2hPG (mmol/l)	4.42 (0.02) ^*^	6.23 (0.02)	4.55 (0.02) ^*^	6.37 (0.01)
Fasting insulin (ρmol/l)	−0.07 (0.03) ^*†^	0.09 (0.03)	−0.13 (0.04) ^*^	0.04 (0.03)
HOMA-IR	−0.04 (0.03)^*†^	0.10 (0.03)	−0.13 (0.04)^*^	0.01 (0.03)
Total serum cholesterol (mmol/l)	6.11 (0.03)	6.06 (0.03)	6.07 (0.03)	6.08 (0.02)
Blood pressure (mmHg)				
Systolic	138 (0.42) ^*^	141 (0.41)	133 (0.59)	135 (0.37)
Diastolic	84 (0.25) ^*^	86 (0.25)	82 (0.32) ^*^	83 (0.20)
Current smoking (%)	32.9 ^*^	23.3	24.8 ^*^	18.9
Hypertension (%)	48.7 ^*^	64.2	45.0 ^*^	51.7
Incidence per1000 person-years (no.)				
CHD	6.7 (225)	7.9 (241)	1.3 (24)	1.9 (91)
IS	2.8 (96) ^*^	4.5 (139)	1.4 (25)	1.7 (81)
CHD + IS	8.7 (293) ^*^	11.3 (345)	2.6 (48)	3.5 (165)

Data are age- and cohort- adjusted means (standard error) or as noted. ^*^p < 0.05 for difference between the two glucose groups in men or in women; ^†^Z-score for 2443 men and 1639 women who had fasting insulin measurements. CHD, coronary heart disease; FPG, fasting plasma glucose; 2hPG, 2-hour plasma glucose; HOMA-IR, homeostasis model assessment of insulin resistance; IS, ischemic stroke.

**Figure 1:**
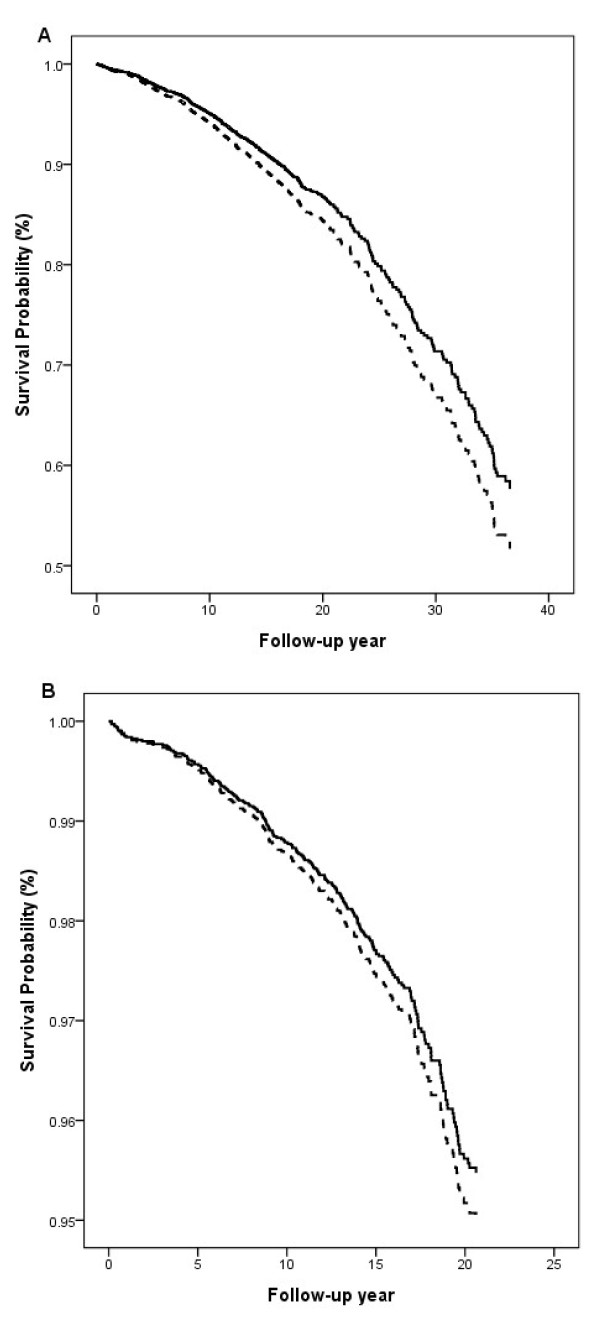
**Survival curves for incidence of composite CVD events derived from Cox regression analysis for Group I (solid line) and Group II (dashed line) in men (A) and in women (B).** The analyses were adjusted for age, cohort, fasting plasma glucose, body mass index, total cholesterol, smoking and hypertension status.

Since the interaction between the two glucose groups and sex was statistically significant (p < 0.001), data analyses were made separately for men and women. Multivariate-adjusted HRs for IS and composite CVD events were significantly higher in Group II than in Group I in men, but not in women (Table 3). The survival profile was better in group I than in group II for composite CVD events in both sexes (Figure 1). Replacing the hypertension status (yes versus no) with continuous systolic blood pressure in the Cox model did not change the results substantially (data not shown). HRs corresponding to a one unit increase in the difference between 2hPG and FPG concentrations (2hPG-FPG) were significantly increased for the CHD events in women and composite CVD events in all individuals (Table 3).

**Table 3:** Hazard ratios and 95% confidence intervals for incidence of coronary heart disease and ischemic stroke

	**CHD**		**IS**		**CHD + IS**	
	**HR**	**95% CI**	**HR**	**95% CI**	**HR**	**95% CI**
Men (n = 3743)						
Group II versus Group I	1.13	0.93-1.37	1.40	1.06-1.85	1.20	1.01-1.42
2hPG-FPG (continuous)	1.03	0.95-1.12	1.11	0.99-1.25	1.07	0.99-1.14
Women (n = 3916)						
Group II versus Group I	1.33	0.83-2.12	0.94	0.59-1.50	1.10	0.79-1.54
2hPG-FPG (continuous)	1.24	1.03-1.50	1.01	0.83-1.23	1.13	0.99-1.30
Total (n = 7659)						
Group II versus Group I	1.15	0.97-1.38	1.27	1.00-1.62	1.18	1.02-1.37
2hPG-FPG (continuous)	1.06	0.98-1.14	1.09	0.98-1.20	1.08	1.01-1.15

Adjusting for age, cohort, body mass index, FPG, total serum cholesterol, smoking status and hypertension status, and sex when men and women combined. CHD, coronary heart disease; 95% CI, 95% confidence interval; FPG, fasting plasma glucose; Group I, 2hPG ≤ FPG; Group II, 2hPG > FPG; 2hPG, 2-hour plasma glucose; 2hPG-FPG, the difference between 2hPG and FPG; HR, hazard ratio; IS, ischemic stroke.

Stratified data analysis was also performed by FPG of ≤ 5.6 mmol/l and 5.6-6.1 mmol/l, and by age of < 60 years and ≥ 60 years for both men and women combined. Multivariate-adjusted HRs (95% CIs) for the composite CVD events for Group II as compared with Group I were 1.09 (0.91-1.30) and 1.41 (1.06-1.86) in the lower and the higher FPG categories; and 1.16 (0.96-1.40) and 1.27 (0.99-1.64), respectively, in the younger and older age groups. In addition, the same data analysis was also made in a subgroup of individuals who had fasting insulin measurements (n = 4082), producing an HR (95% CI) of 1.10 (0.90-1.34) in a model without fasting insulin, to 1.09 (0.89-1.33) when the model fitting with fasting insulin, and to 1.10 (0.90-1.34) when replacing fasting insulin with HOMA-IR. Neither the fasting insulin levels nor the HOMA-IR changed the results substantially. The HR (95% CI) for the composite CVD events was 1.07 (0.89-1.30) and 1.08 (0.89-1.30), respectively in models without and with physical activity and education in a subgroup of participants (n = 5331).

## Discussion

In these prospective cohort studies, 11.0% of the participants who had normoglycemia at baseline developed first CHD or IS events during a median follow-up of 16.4 years. The risk to develop the CHD or IS was increased in people whose 2hPG was higher than whose FPG.

Two-hour plasma glucose concentration is a better predictor than FPG for the incidence of CHD [8,9] and IS [10] among general populations with hyperglycemia. Moreover, post-challenge glucose concentrations contribute to a greater risk on the patients with CHD [28] or IS [29-31] than slightly elevated FPG. In contrast, FPG was better predictor than 2hPG for total mortality and CVD mortality in the CHD patients in another study [32]. A number of studies have shown that IFG and IGT reflect different pathophysiological mechanisms of abnormal glucose homeostasis [33,34]. Fasting hyperglycemia is associated with reduced hepatic insulin resistance and decreased first-phase insulin secretion, while elevated post-challenge glucose concentration is associated with peripheral insulin resistance and impairment of both early- and late-phase insulin response [35,36].

Among individuals with normal glucose range, people whose 2hPG did not return to their FPG levels during a 2-h OGTT had a significantly higher risk for future type 2 diabetes [16], worse cardiovascular risk profile [37] and higher CVD mortality [17] compared with those whose 2hPG were equal to or lower than their FPG levels. This was further confirmed in our current study with incident CVD events. The normoglycemic status is a balance between glucose production and disposal mediated through a synergistic effect of skeletal muscle, pancreas beta cells and liver [38]. Elevated fasting insulin levels have been shown to contribute to the progress to diabetes and CVD among individuals with normoglycemia [16,37]. Our study showed that normoglycemic individuals whose 2hPG did not return to FPG levels had elevated fasting insulin levels and HOMA-IR compared with those who did. Beta-cell dysfunction and insulin resistance have been suggested to be present already in individuals with normal glucose range [11,39]. Both insulin resistance and endothelial dysfunction have been considered as the underlying mechanism contributing to the development of atherosclerosis [40-44]. Other proposed factors include elevated plasma free fatty acids concentrations, which appears to be an early trigger for multiple pathways leading to atherogenesis in non-diabetic healthy subjects [45], and elevated fetuin-A levels which are associated with an increased risk of myocardial infarction and IS in the general population [46]. While these have been reported to be associated with development of CVD in non-diabetic individuals, to what extent these factors contribute to the increased risk of CVD in people with high normal 2hPG is not known, and needs to be further investigated.

The strengths of our study include a relatively long duration of follow-up, a large sample size and a standard 2-h OGTT to define normoglycemia. In this collaborative analysis, all studies were population-based with a random sampling method except for the Helsinki Policemen Study. To take into account the discrepancies between cohorts, the cohort was adjusted as a covariate in the data analysis. The Finnish [25,26] and Swedish [27] Causes of Death Register and Hospital Discharge Register, the source of the data on CHD and IS events in this study, have high coverage and diagnostic accuracy. Our study has also certain limitations. The statistical power of the analysis was relatively lower in women due to small number of incident events. Other known cardiovascular risk factors, such as waist circumference, HbA1c levels and dietary factors were not available for all cohorts included in the current data analysis. Lipid-lowering treatment, types of antihypertensive treatment and hormone replacement therapy for the postmenopausal women were not included in the current database. Furthermore, we did not have any measurements of markers of inflammation and endothelial dysfunction, both of which have been shown to relate more strongly to post-load than to fasting plasma glucose concentrations, and might represent unmeasured confounders which underlie the observed relationships [47,48]. To what extent these missing variables will affect the results is not known and need to be further investigated.

## Conclusion

In the normoglycemic range defined by both 2hPG and FPG criteria, 2hPG after a 75 g OGTT unable to return to the FPG level was associated with worse CVD profile, elevated fasting insulin levels and increased CVD events. The underlying mechanism and clinical implications need to be further investigated.

## Abbreviations

BMI, Body mass index; CHD, Coronary heart disease; 95% CI, 95% confidence interval; CVD, Cardiovascular disease; DECODE, Diabetes Epidemiology: Collaborative analysis Of Diagnostic criteria in Europe; FPG, Fasting plasma glucose; 2hPG, 2-hour plasma glucose; HR, Hazard ratio; IS, Ischemic stroke; OGTT, Oral glucose tolerance test.

## Competing interests

The authors declare that they have no competing interests.

## Authors’ contributions

FN carried out the data analysis and drafted the manuscript. QQ participated in the concept design, funding and coordination of the study and in drafting the manuscript. AO, CDAS, JMD, JSY, JT, KP, LZ and TL contributed to data collection, discussion and approved the final version of the manuscript.
